# Supplementary material for: Concerted and multidisciplinary management of COVID-19 drug therapies during the first two epidemic waves in a tertiary hospital in Marseille, France: Results of the PHARMA-COVID study
Source: PLoS One. 2023 Mar 17;18(3):e0283165. doi: 10.1371/journal.pone.0283165 (PMC10022761; doi:10.1371/journal.pone.0283165)
Supplement: S1 Table — (DOCX) [file pone.0283165.s001.docx]

**Table S1:** Characteristics and outcomes of patients with (ST+) or without (ST-) anti-COVID-19 specific therapies (excluding anticoagulant) during the first (W1) and second (W2) epidemic waves.

|  | Global | Specific anti-COVID-19 therapies (ST) | | ST+ during epidemic waves | | ST+  vs ST-  P-value* | ST+ W1  vs ST+ w2 P-value* |
| --- | --- | --- | --- | --- | --- | --- | --- |
|  |  | Yes  (ST+) | No  (ST-) | ST+  during W1 | ST+  during W2 |  |  |
| **Number of patients hospitalized for COVID-19** | 607 | 372 | 235 | 154 | 218 |  |  |
|  |  |  |  |  |  |  |  |
| **Patients characteristics** (n= 607) |  |  |  |  |  |  |  |
| sex ratio (M/F) | 1.7 | 1.9 | 1.4 | 1.9 | 1.9 | 0.148 | 1 |
| mean age ± SD (years) |  | 65.6 ± 15.2 | 63.4 ± 17.6 | 64.1 ± 15.6 | 66.7 ± 14.8 | 0.115 | 0.1 |
| mean BMI ± SD (kg/m2) (n= 529) | 27.6 ± 5.8 | 28 ± 6 | 26.7 ± 5.4 | 27.4 ± 5.3 | 28.4 ± 6.4 | 0.015 | 0.107 |
| mean modified Charlson index ± SD | 1.3 ± 2.2 | 1.3 ± 2.2 | 1.3 ± 2.3 | 1.3 ± 2.3 | 1.3 ± 2.1 | 0.924 | 0.797 |
| obesity (%) (n= 531) | 147 (28%) | 107 (30%) | 40 (22%) | 43 (31%) | 64 (30%) | 0.056 | 0.918 |
| diabetes (%) | 203 (33%) | 136 (37%) | 67 (29%) | 57 (37%) | 79 (36%) | 0.05 | 0.965 |
| high blood pressure (%) | 259 (43%) | 172 (46%) | 87 (37%) | 70 (45%) | 102 (47%) | 0.031 | 0.882 |
|  |  |  |  |  |  |  |  |
| **Patients outcome** (n= 607) |  |  |  |  |  |  |  |
| ICU admission (%) | 134 (22%) | 120 (32%) | 14 (6%) | 38 (25%) | 82 (38%) | <0.001 | 0.012 |
| average length of stay ± SD (days) | 13.3 ± 16 | 16.8 ± 18.3 | 7.9 ± 9.1 | 14.8 ± 16.1 | 18.2 ± 19.5 | <0.001 | 0.069 |
| mortality rate (%) | 92 (15%) | 56 (15%) | 36 (15%) | 19 (12%) | 37 (17%) | 1 | 0.278 |
| mean age at death ± SD (years) (n= 92) | 71.5 ± 16.3 | 74.3 ± 12.2 | 67.3 ± 20.4 | 71.7 ± 11.9 | 75.6 ± 12.1 | 0.073 | 0.268 |
| SD, standard deviation ; BMI, body mass index  * Chi-square was used for categorical variables and the Student's t test was used for quantitative variables | | | | | | | |
